# Advancements in consolidative thoracic radiotherapy following first-line immunotherapy in conjunction with chemotherapy for extensive-stage small cell lung cancer

**DOI:** 10.3389/fimmu.2025.1665072

**Published:** 2025-11-03

**Authors:** Jiang Liu, Jianhua Liu, Dadong Chen, Yan Zhu, Xiang Wu, Yin Cai

**Affiliations:** ^1^ Department of Oncology, Xinghua People’s Hospital Affiliated to Yangzhou University, Xinghua, Jiangsu, China; ^2^ Department of Otorhinolaryngology, Xinghua People’s Hospital Affiliated to Yangzhou University, Xinghua, Jiangsu, China; ^3^ Department of Oncology, Dongtai Hospital of Traditional Chinese Medicine, Dongtai, Jiangsu, China

**Keywords:** small cell lung cancer, ES-SCLC, immune checkpoint inhibitors, immunotherapy, chemotherapy, thoracic radiotherapy

## Abstract

Small cell lung cancer (SCLC) presents challenges due to its high invasiveness and rapid progression, resulting in an inferior prognosis. Approximately 70% of patients have developed an extensive stage at the time of diagnosis. While most patients with extensive-stage SCLC (ES-SCLC) are sensitive to chemotherapy, they remain at high risk of local recurrence and distant metastasis in the short term. In the era of chemotherapy, studies have indicated the potential survival benefits of consolidative thoracic radiotherapy (cTRT) for patients responding to systemic treatment. The introduction of immunotherapy has significantly transformed the treatment landscape for SCLC. The combination of immune checkpoint inhibitors (ICIs) with chemotherapy has emerged as the new standard for first-line treatment of ES-SCLC. Nevertheless, controversy surrounds the role of cTRT after the first-line treatment of ES-SCLC in the context of immunotherapy, especially considering advancements in imaging staging methods and precise radiotherapy technology. This review focuses on the application value and latest research advancements in cTRT following first-line immunotherapy combined with chemotherapy in ES-SCLC, providing valuable insights for clinical practice.

## 1 Introduction

SCLC represents the most prevalent primary neuroendocrine tumor of the lung and is recognized as one of the most lethal malignancies, characterized by a notably low 5-year survival rate (1). SCLC exhibits unique biological characteristics, characterized by high invasiveness, rapid growth, high sensitivity to radiotherapy and chemotherapy, and a tendency for early distant metastasis. Approximately 70% of patients are initially diagnosed with extensive stage (2). Over the past few decades, platinum-based chemotherapy has been the standard first-line treatment regimen for SCLC (3, 4). Despite the tumor’s initial sensitivity to chemotherapy, the disease is characterized by a high recurrence rate and a meager response to second-line treatment, resulting in a median overall survival (OS) of less than one year (4, 5). Radiotherapy is a pivotal component in the comprehensive management of SCLC. Previous studies (6–8) have demonstrated that cTRT administered to patients who respond well to systemic therapy yields a high degree of local disease control and extends overall survival within the context of the chemotherapy era.

Currently, the predominant immune checkpoint inhibitors (ICIs) in clinical practice include programmed death receptor 1/programmed death-ligand 1(PD-1/PD-L1) inhibitors and cytotoxic T-lymphocyte antigen-4(CTLA-4) inhibitors. PD-1/PD-L1 inhibitors restore the killing function of T cells against tumor cells by impeding the binding of PD-L1 on the surface of tumor cells to PD-1 on the surface of T cells, thereby enhancing anti-tumor immune responses. CTLA-4 inhibitors competitively bind to ligand B7 with CTLA-4, relieving the inhibitory factors that impede T cell activation, thereby exerting an anti-tumor effect (9). In recent years, multiple clinical studies (10–13) have demonstrated that first-line standard chemotherapy combined with immunotherapy can significantly prolong the survival of ES-SCLC patients, establishing its position as a first-line treatment for small cell lung cancer. The value of cTRT in the era of immunotherapy is still controversial. In this review, we summarized the advancements in research concerning immunotherapy and cTRT for ES-SCLC and explored the application value of cTRT after first-line immunotherapy combined with chemotherapy in ES-SCLC.

## 2 Immunotherapy in ES-SCLC

### 2.1 Advancements in immunotherapy for ES-SCLC

Tumor cells can utilize immune checkpoints to evade recognition and clearance by the immune system. ICIs work by initiating a series of immune responses to restore anti-tumor immunity, achieving the goal of recognizing and killing tumor cells. Therefore, blocking PD-1/PD-L1 and CTLA-4 with ICIs to restore the body’s anti-tumor immune function is an effective measure for treating ES-SCLC.

Clinical trials of ICIs for SCLC started with CTLA-4 inhibitors. A phase II clinical trial (14) investigated the efficacy and safety of chemotherapy combined with ipilimumab in treating ES-SCLC. The results showed that compared with the chemotherapy group, the ipilimumab combined with chemotherapy group had no significant benefit in progression-free survival (PFS) and OS, but increased immune-related toxicity. Moreover, another large phase 3 confirmatory trial (15) demonstrated that ipilimumab in combination with chemotherapy failed to extend OS versus chemotherapy alone in ES-SCLC patients.

The IMpower 133 trial (10) evaluated the efficacy and safety of the combination of atezolizumab with chemotherapy as first-line treatment for ES-SCLC. The median PFS and OS in the atezolizumab group were significantly longer than those in the placebo group, and there was no statistically significant difference in the incidence of adverse events between the two groups. The CASPIAN study (11, 16) demonstrated that median OS was significantly improved in the durvalumab group (13.0 months [95% CI 11.5-14.8]) compared with the placebo group (10.3 months [95% CI 9.3-11.2]). Adebrelimab is a Chinese-made humanized anti-PD-L1 monoclonal antibody. In the CAPSTONE-1 study (13), the combination of adebrelimab with chemotherapy significantly prolonged median OS compared with chemotherapy alone in ES-SCLC patients. Based on the findings of the IMpower 133 and CASPIAN studies, atezolizumab/durvalumab combined with etoposide and carboplatin/cisplatin has been approved by the United States Food and Drug Administration (FDA) and the National Medical Products Administration (NMPA) in China as the preferred regimen for first-line treatment for ES-SCLC. In addition, the results of CAPSTONE-1 provide strong evidence for the combination of adebrelimab and chemotherapy as first-line treatment for ES-SCLC, and adebrelimab has also been approved by NMPA as a new option.

Nevertheless, the findings of PD-1 inhibitors exhibit considerable variability in SCLC patients. A pooled analysis (17) from KEYNOTE-028 and KEYNOTE-158 indicated durable antitumor activity of pembrolizumab in patients with relapsed or metastatic SCLC who had received two or more prior lines of therapy, irrespective of PD-L1 expression. Based on the results of the lung cancer cohorts from the KEYNOTE-028 and KEYNOTE-158 studies, the FDA granted accelerated approval for pembrolizumab monotherapy for relapsed ES-SCLC after two or more lines of therapy on June 17, 2019. KEYNOTE-604 study (18) investigated the efficacy and safety of pembrolizumab combined with chemotherapy as a first-line treatment for ES-SCLC. The findings showed that compared with chemotherapy alone, pembrolizumab in combination with chemotherapy significantly improved PFS but not OS, and there was no statistically significant difference in the incidence of adverse events between the two groups. Pembrolizumab was withdrawn in 2021 due to the confirmatory phase 3 clinical KEYNOTE-604 study only achieving PFS among its primary endpoints, but not a statistically significant OS endpoint. A sustained anti-tumor response was observed in nivolumab as at least third-line therapy for recurrent SCLC (19). Based on this result, nivolumab was approved by the FDA for at least third-line therapy of recurrent SCLC in August 2018. Unfortunately, nivolumab was also withdrawn in December 2020 due to the disappointing results of the following studies. CheckMate 331 study (20) showed that nivolumab did not improve OS compared with chemotherapy in the second-line treatment of relapsed SCLC. CheckMate 451 trial (21) indicated that maintenance therapy with nivolumab in combination with ipilimumab failed to extend OS for ES-SCLC patients who responded to first-line treatment. The failure of pembrolizumab and nivolumab in treating SCLC does not imply that all PD-1 inhibitors are insensitive to this cancer. Serplulimab is a humanized anti-PD-1 monoclonal antibody. The ASTRUM-005 study (12) showed that first-line treatment with serplulimab plus chemotherapy significantly improved PFS and OS compared with chemotherapy alone in ES-SCLC, while the incidence of adverse events did not differ significantly between the two groups. Serplulimab has become the world’s first PD-1 inhibitor for ES-SCLC based on these findings. Clinical trials changing the first-line treatment strategy for ES-SCLC are displayed in **Table 1**.

**Table 1:** Clinical trials changing the first-line treatment strategy for ES-SCLC.

Trial	Population	Treatment arms	Primary endpoint	ORR%	mPFS (months)	mOS (months)
IMpower133 (10) Randomized Phase 3, N = 403	Untreated ES-SCLC	Atezolizumab plus CT vs. CT	PFS and OS	60.2 vs. 64.4 (p NR)	5.2 vs. 4.3 (HR = 0.77, P = 0.02)	12.3 vs. 10.3 (HR = 0.70, P = 0.007)
CASPIAN (11, 16) Randomized Phase 3, N = 537	Untreated ES-SCLC	Durvalumab plus CT vs. CT	OS	68 vs. 58 (p NR)	5.1vs.5.4 (HR = 0.78, p NR)	12.9 vs. 10.5 (HR = 0.71, p = 0.0003)
ASTRUM-005 (12) Randomized Phase 3, N=585	Untreated ES-SCLC	Serplulimab plus CT vs. CT	OS	80.2 vs. 70.4 (p NR)	5.7 vs. 4.3 (HR = 0.48, p NR)	15.4 vs. 10.9 (HR = 0.63, p< 0.001)
CAPSTONE-1 (13) Randomized Phase 3, N=462	Untreated ES-SCLC	Adremelimab plus CT vs. CT	OS	70.4 vs. 65.9 (p NR)	5.8 vs 5.6 (HR = 0.67, p< 0.0001)	15.3 vs. 12.8 (HR = 0.72, p = 0.0017)

ES-SCLC, extensive stage small-cell lung cancer; ORR, overall response rate; N, number; vs., versus; mPFS, median progression-free survival; mOS, median overall survival; CT, chemotherapy; HR, hazard ratio; NR, not reported.

### 2.2 Biomarkers for immunotherapy in SCLC

Although immunotherapy has significantly prolonged the survival of patients with ES-SCLC, the proportion of those who benefit in the real world remains limited. Therefore, it is necessary to screen biomarkers predicting the efficacy of ICIs. Biomarkers that have predictive value for immunotherapy in NSCLC do not apply to SCLC. PD-L1 expression did not predict response to chemotherapy in combination with ICIs therapy in several previous studies (18, 21, 22). Furthermore, a meta-analysis (23) enrolling 2792 patients with SCLC indicates that PD-L1 expression was a favorable but not statistically significant prognostic factor. The reasons for the discrepancy in the predictive value of PD-L1 expression in NSCLC and SCLC immunotherapy may be as follows. On one hand, unlike NSCLC, PD-L1 expression is generally low in SCLC (24). On the other hand, due to tumor heterogeneity and limitations of PD-L1 detection methods, biopsy samples cannot fully reflect the expression of PD-L1 in SCLC tissues.

Besides PD-L1 expression, tumor mutation burden (TMB) is another important marker for predicting the response to immunotherapy. CheckMate 032 study (25) explored the predictive value of TMB and found that high TMB was associated with a superior response in the ipilimumab combined with nivolumab group but not the nivolumab monotherapy group. The role of TMB was also studied in the CASPIAN trial (26), evaluating durvalumab in combination with platinum-etoposide (EP) versus EP alone as first-line therapy in ES-SCLC. This research indicated that tissue TMB (tTMB) status was not associated with OS benefit. In exploratory biomarker analysis of the phase 3 KEYNOTE-604 study (27), an inferior OS was observed in the experimental compared with the control arm in the high TMB (>175 mut/exome) subgroup. Therefore, the predictive potential of TMB for immunotherapy efficacy in SCLC is still lacking evidence and needs to be further verified by subsequent studies.

In recent years, researchers have classified SCLC into four subtypes: SCLC-A, SCLC-N, SCLC-Y, and a distinct subtype of SCLC with lower expression of all three transcription factors, based on the differential expression of key transcriptional regulatory factors such as achaete-scute homologue 1 (ASCL1), neurogenic differentiation factor 1 (NeuroD1) and POU class 2 homeobox 3 (POU2F3) (28). Since the fourth subtype is characterized by expression of inflammatory genes, this subtype was named SCLC-Inflamed, or SCLC-I (28). The SCLC-I subtype benefits more from immunotherapy than the other three subtypes (28). An exploratory analysis from the IMpower133 trial indicated that a higher proportion of patients with long-term survival in both groups had SCLC-I subtype, which was more pronounced in the atezolizumab group (29). The SCLC-I subtype has potential predictive value for SCLC immunotherapy response and deserves further investigations to explore the related mechanisms. In addition, scholars have also explored the correlations between the activation of cellular signaling pathways (30) and the characteristics of the tumor immune microenvironment (31–34) with the efficacy of immunotherapy for SCLC, providing novel insights for subsequent research. In summary, accurately screening the populations that benefit most from immunotherapy is an urgent task and challenge.

## 3 cTRT for ES-SCLC in the era of chemotherapy

Over the past few decades, platinum-based chemotherapy has been recognized as the standard first-line treatment for ES-SCLC, yielding a median survival of approximately ten months (35). Nevertheless, approximately 70% to 90% of patients exhibit residual intrathoracic disease following chemotherapy, and progression to refractory disease typically transpires within the first year after initial treatments (36, 37). Therefore, the localized management of intrathoracic residual lesions is crucial for postponing disease progression and ensuring long-term survival. The feasibility of cTRT in ES-SCLC is partly due to the heightened radio-sensitivity of SCLC. Its clinical impact has been previously documented in both prospective and retrospective studies.

### 3.1 The application value of cTRT in ES-SCLC

As early as 1999, Jeremic et al. first confirmed the value of thoracic radiotherapy in patients with ES-SCLC who achieved complete/partial remission (CR/PR) after initial chemotherapy (6). Their results showed that cTRT after chemotherapy could increase the 5-year OS rate by 5.4% (9.1% vs. 3.7%, P = 0.041). In 2007, the European Organization for Research and Treatment of Cancer (EORTC) reported that approximately 75% of patients retained intrathoracic residual lesions post-chemotherapy, and nearly 90% of these patients experienced an intrathoracic recurrence within a year, indicating a potential benefit from thoracic radiotherapy (36). The CREST study (7) in 2015 encompassed 495 ES-SCLC patients who responded effectively to 4–6 cycles of platinum-based chemotherapy. These patients were then randomly assigned to receive cTRT (30Gy in 10fractions) or no cTRT. The primary endpoint was the difference in the one-year OS rate between the groups, which was not statistically significant (33% vs. 28%, HR = 0.84, P = 0.066). However, upon secondary analysis of the CREST study, it was observed that the cTRT group had a 10% increase in the 2-year OS rate (13% vs. 3%, P = 0.004) and improved median PFS (4 months vs. 3 months, P = 0.001). Additionally, thoracic radiotherapy significantly decreased the intrathoracic recurrence rate (79.8% vs. 43.7%, P < 0.0001). Although this study establishes the role of cTRT in ES-SCLC, it also raises several questions for future research. First, despite cTRT, the local recurrence rate remains alarmingly high at over 40%, suggesting that the radiotherapy dose may be inadequate for durable control. Second, a mere 13% of patients underwent brain CT/MRI evaluation post-chemotherapy, potentially overlooking those who had progressed. Third, in both the cTRT and non-cTRT groups, 66.4% and 43.9% of patients experienced extrathoracic progression. Could enhanced control of extrathoracic lesions enhance the benefits of cTRT? In addition, the phase II randomized controlled study, RTOG-0937, assessed the efficacy of prophylactic cranial irradiation (PCI) and the combination of PCI with consolidative radiation therapy (PCI+cRT) in treating intrathoracic disease and extracranial metastases for ES-SCLC (38). This study was discontinued in the interim analysis due to invalid OS in both groups. Despite no significant difference in one-year OS rates, the consolidation radiotherapy group demonstrated a lower locally regional recurrence rate (25.8% vs. 62.5%) and a significantly extended median PFS (4.9 months vs. 2.9 months, p=0.01). The insufficient OS benefit may be attributed to a higher number of patients with higher tumor burden, lower CR rates, and poorer performance status (PS) within the consolidation radiotherapy group, indicating significant selection bias. The use of PCI in patients with SCLC remains a topic of controversy. Risk factors for the development of brain metastases are largely unidentified, which hinders the development of personalized treatment strategies. A recent meta-analysis (39) indicated that younger age, higher T-stage, and extensive disease (ED) are risk factors for brain metastases, suggesting that PCI should be emphasized in these patients. What’s more, a retrospective study showed that neurotoxicity associated with PCI tends to occur more frequently in older patients (40). Therefore, it is reasonable to consider that PCI is not recommended for older patients and those with poor Karnofsky Performance Status (KPS).

Recently, retrospective studies have further investigated the role of cTRT. In 2019, Deng et al. performed a retrospective analysis of the potential value of thoracic intensity-modulated radiation therapy (IMRT) in patients with ES-SCLC who responded to platinum-based chemotherapy (41). The findings revealed a significant reduction in the local recurrence rate in the group receiving cTRT compared with the chemotherapy alone (19.2% vs.75.6%, P = 0.001), and the 5-year OS rate increased by 8.7% (12.3% vs. 3.6%, P<0.001). A meta-analysis (42) also confirmed that cTRT can provide survival benefits to ES-SCLC patients who respond effectively to first-line treatments, while maintaining an overall acceptable adverse reaction profile. Prospective and retrospective trials of TRT for ES-SCLC in the chemotherapy era are displayed in **Table 2**.

**Table 2:** Prospective and retrospective trials of TRT for ES-SCLC in the chemotherapy era.

Trial	Year	Groups	TRT dose	PFS (months)	OS (months)	Adverse Reaction
Jeremic et al. (6) Randomized Phase 3, N = 109	1999	TRT plus CHT vs. CHT	54Gy/36f (1.5Gy bid)	mLRFS: 30 vs. 22 (p NR), 5-year LRFS: 20% vs. 8.1% (P = 0.062)	mOS: 17 vs. 11 (p NR), 5-year OS: 9.1% vs. 3.7% (P = 0.041).	Acute grade 3 or higher AEs were lower (p NR)
CREST trial (7) Randomized Phase 3, N = 495	2015	TRT vs. Non-TRT	30Gy/10f	mPFS: 4 vs. 3 (p NR), 6-month PFS: 24% vs. 20% (P = 0.001)	mOS: 11.8 vs. 8.3 (p<0·0001), 2-year OS: 13% vs. 3% (P = 0.004).	Grade 3 or higher AEs: 10.5% vs. 7.3% (P = 0.28)
RTOG-0937 (38) Randomized Phase 2, N = 86	2017	PCI plus cRT* vs. PCI	45Gy/15f	mPFS: 4.9 vs. 2.9 (P = 0.01), one-year rates of progression: 75.0% vs. 79.6% (P = 0.01)	mOS: 13.8 vs. 15.8 (P = 0.21), 1-year OS: 50.8% vs. 60.1% (p NR)	Grade-3 or greater AEs: 36.4% vs. 23.8% (P = 0.24)
Deng et al. (41) Retrospective, N = 144	2019	TRT plus CHT vs. CHT	32-67Gy/25-33f	5-year PFS rate: 4.3% vs. 0.0%, (P = 0.023)	5-year OS rate: 10.5% vs. 1.6% (P < 0.001)	The rates of ≥ 2 grade pneumonitis and esophagitis were 28.5% and 12.3% with TRT
Zhang et al. (46) Retrospective, N = 305	2019	TRT plus CHT vs. CHT	40–60Gy /20–30; 30–45Gy /10–15	mPFS:14.5vs.10.1 ( p=0.006); for oligometastasis,mPFS:16.5vs.9.1( p=0.005)	mOS:17.8 vs.16.5 (p=0.061); for oligometastasis,mOS:19.2 vs.15.6( p=0.039	pneumonitis and esophagitis of any grade were 27.6% and 14.3% with TRT
Han et al. (50) Retrospective, N = 492	2021	TRT plus CHT vs. CHT	40–66Gy, 1.8–2Gy/f; 45Gy, 1.5Gy bid; 30–51Gy, 3Gy/f	mPFS:9.3 vs. 6.0( p< 0.001), mLRFS:12.0 vs. 6.6( p< 0.001)	mOS:18.1 vs. 10.8 ( p< 0.001)	The rates of ≥ 2 grade pneumonitis and esophagitis in high-dose were 25% and 18% with TRT

TRT, thoracic radiotherapy; mOS, median overall survival; mPFS, median progression-free survival; CHT, chemotherapy; LRFS: local recurrence-free survival; AEs, adverse events; PCI, prophylactic cranial irradiation; cRT, consolidative radiation therapy ; bid, twice daily; NR, not reported; PCI plus cRT*,PCI plus consolidative radiation therapy to intra -thoracic disease and extracranial metastases.

### 3.2 Screening for beneficial populations in cTRT

ES-SCLC represents a group of highly heterogeneous diseases, necessitating the screening of populations that may benefit from cTRT. The CREST trial revealed that approximately 88% of patients possess intrathoracic remnants, with these individuals demonstrating a higher likelihood of benefiting from cTRT (43). In advanced NSCLC, the application of radical radiotherapy to both primary and metastatic lesions can confer survival advantages to patients with oligometastases (44). However, a contentious debate persists concerning both the definition of oligometastases and the potential benefits of localized treatment for patients with ES-SCLC. The secondary analysis of the CREST study (45) revealed that patients with fewer than or equal to two metastatic sites exhibited superior OS and PFS compared with those with more than three metastases, irrespective of cTRT treatment. Furthermore, patients with fewer than or equal to two metastatic sites who received cTRT demonstrated a significant improvement in PFS (p = 0.003). In a retrospective investigation (46), oligometastases were defined as single organ or non-regional lymph node metastasis, contiguous vertebral metastasis amenable to safe inclusion in the radiotherapy plan, or multiple brain metastases suitable for whole brain radiotherapy. The findings indicated that thoracic radiotherapy could enhance OS in patients with oligometastases, whereas patients with liver, brain or multiple metastases did not benefit from thoracic radiotherapy. Another retrospective study (47) also suggested that thoracic radiotherapy could improve the OS rate for patients with isolated metastasis. In summary, published evidence suggests that patients most likely to derive a significant survival benefit from cTRT are those with a good response to initial systemic therapy (CR/PR), limited extrathoracic disease burden (e.g. oligometastatic disease, defined as ≤ double metastatic sites or isolated metastasis), and the absence of high-risk metastatic sites such as the liver and brain. Conversely, patients with extensive metastatic involvement (e.g. > three sites, liver metastases) or rapid systemic progression are less likely to benefit from aggressive local consolidation. The evidence from retrospective analyses is inherently limited by their non-randomized design and potential for confounding factors.

### 3.3 Exploration of dose fractionation model

An early prospective investigation has demonstrated that a total dose of 54Gy in 36 fractions, administered twice daily, is both safe and effective for patients who achieve CR or PR following chemotherapy (6). In the CREST study (7), a palliative dose of 30Gy, administered in ten fractions, was utilized for thoracic radiotherapy, yet the local recurrence rate was as high as 43.7%, suggesting that the local dose may be insufficient. The RTOG 0937 study (38) recommended a consolidation radiotherapy dose of 45Gy in 15 fractions, resulting in a lower local regional recurrence rate (first site of recurrence) than that observed in the CREST study (25.8%). The multicenter, prospective, observational TRENDS study (48) evaluated the patterns of consolidative chest radiotherapy (including techniques, target volumes, and dosing) as well as its effectiveness in disease control and tolerability in patients with ES-SCLC. The study confirmed that consolidative chest radiotherapy is effective in reducing the risk of intrathoracic disease progression and is well-tolerated in extensive-stage SCLC patients who respond favorably to first-line chemotherapy. However, there is currently a lack of large-sample prospective studies to compare the benefits of different dose fractionation modes in ES-SCLC. Some retrospective studies suggest that increasing the dose of thoracic radiotherapy may confer survival benefits. One retrospective study (49) demonstrated that patients with a thoracic radiotherapy dose of ≥45Gy had a significantly better prognosis than those with a dose <45Gy (2-year OS rate was 25.2% vs.15.1%, P<0.001). Another retrospective study (50) found that increasing the dose of thoracic radiotherapy (time-adjusted biologically effective dose >50Gy) for patients who achieved CR/PR after chemotherapy did not improve prognosis, and the incidence of grade 2 or higher radiation esophagitis increased. Furthermore, compared with the conventional fractionation of 60Gy in 30 fractions, once daily, the over-fractionation mode of 45Gy in 30 fractions, twice daily, had a longer OS (P = 0.036).

In summary, cTRT remains a recommended treatment for patients with ES-SCLC who have been effectively treated with systemic therapy. Patients with residual intrathoracic lesions may benefit from thoracic radiotherapy. However, the optimal dose fractionation pattern continues to be a subject of debate.

## 4 cTRT for ES-SCLC in the era of immunotherapy.

### 4.1 Theoretical basis of cTRT

The prevailing consensus posits that radiotherapy can remodel the tumor immune microenvironment (51). In addition to the direct damage to tumor cells, radiotherapy can also promote the release of tumor-specific antigens, enhance the immunogenicity of tumor cells, regulate signal transduction, alter the inflammatory tumor immune microenvironment, activate adaptive immune responses, and induce immune-mediated anti-tumor effects within or outside the irradiated field, namely the “immune memory effect” and the “abscopal effect” (52). Furthermore, the effect can be significantly enhanced when radiotherapy is integrated with immune checkpoint inhibitors (ICIs), transforming an ineffective immune response into a robust and enduring one (53). However, radiotherapy may also cause immunosuppressive effects by up-regulating the expression of PD-L1 and inhibiting T-cell activation (54). The immunomodulatory impact of radiotherapy is associated with its fractionation pattern. Research has indicated that low-dose radiotherapy, ranging from 0.5 to 2Gy per fraction, could stimulate innate and adaptive immunity. It also promotes the activation and infiltration of immune effector cells, such as CD4^+^ and CD8^+^ T cells, and restructures the tumor microenvironment within the so-called “immune desert” (55). Compared with traditional fractionation, single high-dose radiotherapy can directly kill tumor cells and more effectively stimulate anti-tumor immune responses by promoting the release of tumor-associated antigens (56). However, this high dose can also lead to vascular damage, induce radioresistance due to tumor hypoxia, and activate DNA exonuclease Trex1, which has a negative immunomodulatory effect (56). The impact of radiotherapy on the immune microenvironment is bidirectional, with the outcome influenced by various factors such as radiation fractionation patterns, dose, mode of immunotherapy, and tumor type.

### 4.2 Investigation into the impact of cTRT on survival

Despite the significant improvement in survival for ES-SCLC patients treated with first-line chemotherapy and ICIs compared with chemotherapy alone, only 0.8% to 2.5% of patients achieved CR (10). Neither the IMPOWER133 nor the CASPIAN studies explored the potential value of cTRT after treatment with the combination of chemotherapy with ICIs. Consequently, there remains controversy over the safety and benefits of cTRT in the context of immunotherapy. Updated data from IMPOWER133 revealed that over 90% of patients had local residual lesions following first-line treatment, including progression of the primary lung lesion in 34% of cases, suggesting that local consolidation therapy may have potential benefits during immunotherapy (22).

Recent studies have explored the potential values of cTRT in the context of first-line chemotherapy and immunotherapy. A retrospective study (57) involving ES-SCLC patients found that those who received cTRT following first-line chemotherapy in combination with PD-L1 monoclonal antibody therapy had a one-year OS rate of 80.2%. Furthermore, only 8% of these patients developed grade 1 or 2 radiation pneumonia. The biologically effective dose of thoracic radiotherapy ranged from 52 to 113Gy, providing preliminary evidence for the feasibility of this therapy for patients who respond effectively to first-line chemotherapy and immunotherapy. Another retrospective study (58), which included 211 patients who underwent first-line chemotherapy and PD-L1 antibody therapy, demonstrated that the group receiving cTRT had a significantly longer OS (median OS: 24.1 months vs.18.5 months, P = 0.016). It is important to note that in both studies, over 80% of the patients who received thoracic radiotherapy had ≤2 metastatic sites. This suggests that patients with a limited extrathoracic tumor burden are more likely to benefit from cTRT. Xie et al. observed that after first-line chemotherapy in conjunction with ICIs for ES-SCLC patients, the cTRT group exhibited a significant improvement in both PFS (8.0 months vs.5.9 months, HR = 0.64, p = 0.025) and OS (22.7 months vs.14.7 months, HR = 0.52, p = 0.015) (59). These findings align with those of two other studies (60, 61). However, a small-sample retrospective study (62) indicated that cTRT following first-line chemotherapy and immunotherapy did not yield a survival benefit (median PFS: 9.1 vs.8.8 months, P = 0.93, median OS: 21.8 vs.24.3 months, P = 0.63), which may be attributed to the higher rate of extrathoracic progression in the cTRT group (91.4% vs. 67.6%, P = 0.02). Since the studies above are all small-sample retrospective studies, they lack sufficient evidence-based support.

At the 2023 annual meetings of the American Society of Clinical Oncology (ASCO), a report was presented on the efficacy and safety of first-line adebrelimab combined with chemotherapy, followed by thoracic radiotherapy for patients with ES-SCLC (63). The findings indicated that the objective response rate (ORR) for patients who underwent thoracic radiotherapy reached 80%, with a median PFS of 7 months. Additionally, grade 3-4 pneumonia occurred in 6% of patients. The conflicting results from these retrospective studies underscore the significant heterogeneity in patient selection, radiotherapy dosing, and patterns of failure. This highlights a critical selection bias. Overall, the current evidence base for combining cTRT with chemo-immunotherapy consists solely of retrospective data with small sample sizes, significant methodological heterogeneity, and potential for confounding. This precludes definitive conclusions regarding efficacy and necessitates validation from prospective randomized trials. Currently, several prospective studies such as NCT05544149, NCT05552846, RAPTOR/NRG, and LU007/NCT04402788, are exploring the potential value and safety of cTRT after first-line chemotherapy in combination with immunotherapy for ES-SCLC. Studies of TRT for ES-SCLC in the immunotherapy era are displayed in **Table 3**.

**Table 3:** Studies of TRT for ES-SCLC in the immunotherapy era.

Trial	Year	Groups	TRT dose	PFS (months)	OS (months)	Adverse Reaction
Li et al. (57) Retrospective, N = 36	2023	TRT+CHT+ICIs	60Gy/28f; 37.5-50Gy/4-5f; 45Gy/15f; 45Gy/30f, bid	mPFS:12.8, 6-month PFS:97.3%, 1-year PFS:53.4%, 2-year PFS:17.9%	mOS: not reached, 6-month OS:97.1%, 1-year OS:80.2%, 2-year OS:53.3%	Most AEs were tolerable and self-limiting
Peng et al. (58) Retrospective, N = 211	2023	TRT+CHT+ ICIs vs. CHT+ ICIs	30Gy/10f; 45Gy/15f; 50Gy/25f; 60Gy/30f	mPFS: 9.5 vs. 7.2 (P = 0.009), one-year PFS rates : 31.6% vs. 14% (P = 0.026)	mOS: 24.1 vs. 18.5 (P = 0.016), one-year OS rates : 73.7% vs. 59.7% (P = 0.072)	pneumonitis : 35.1% vs.15.8%, most were grade 1-2
Xie et al. (59) Retrospective, N = 118	2023	TRT+CHT+ ICIs vs. CHT+ ICIs	30Gy-60Gy/ 2Gy-3Gy	mPFS: 8 vs.5.9 (P = 0.025), one-year PFS rates : 45.2% vs. 16.4% (P NR)	mOS: 22.7 vs. 14.7 (P = 0.015), one-year OS rates : 77.7% vs. 63.0% (P NR)	Grade-3 or greater AEs:37.7% vs.32.8% ( p = 0.58)
Wu et al. (60) Retrospective, N = 22	2022	TRT+CHT+ ICIs vs. CHT+ ICIs	28-64Gy	mPFS: NR vs.6	mOS: not-reached vs. 9.6 (P <0.001)	Most AEs were grade 1-2
Daher et al. (61) Retrospective, N = 126	2022	TRT+CHT+ ICIs vs. CHT+ ICIs	Median dose was 38Gy (24-60Gy)	mPFS: 8.5 vs.5.6 ( p <0.003)	mOS: 27.7 vs. 13.2 (P <0.007)	No pneumonitis and grade 4 or great AEs in cTRT group
Li et al. (62) Retrospective, N = 100	2023	TRT+CHT+ ICIs vs. CHT+ ICIs	30Gy/10f; 45Gy/15f; 50Gy/25f; 60Gy/30f	mPFS: 9.1 vs.8.8 ( P=0.93)	mOS: 21.8 vs. 24.3 (P=0.63)	grade 1-2 pneumonitis: 19.2% ; grade 3 pneumonitis:10.6%
Chen et al. (63) N = 63	2023	TRT+CHT+ ICIs vs. CHT+ ICIs	≥3Gy*10f; ≥2Gy*25f	mPFS: 7	mOS: NR	3-4 grade pneumonia: 6%

TRT, thoracic radiotherapy; ICIs , immune checkpoint inhibitors; mOS, median overall survival; mPFS, median progression-free survival; CHT, chemotherapy; AEs, adverse events; bid, twice daily; NR, not reported.

The optimal timing for cTRT intervention remains a crucial and unresolved issue. Some scholars contend that early cTRT, administered within three cycles of chemotherapy, does not confer significant survival benefits compared with cTRT administered later, after three cycles (64). However, another study indicates that initiating TRT within six cycles of chemotherapy can lead to improved local control rates (50). For patients with ES-SCLC, we believe that systemic therapy is of utmost importance. Local cTRT may be more effectively administered after the disease has been adequately controlled and stabilized, rather than too early in the treatment process. Additionally, the optimal sequence for cTRT and maintenance immunotherapy remains uncertain. According to current guidelines from the American Society of Clinical Oncology, cTRT should be administered within six to eight weeks following chemotherapy and before starting maintenance immunotherapy. It is posited that delivering cTRT during the maintenance immunotherapy phase is a promising treatment strategy. Available evidence indicates that this approach is well-tolerated and is associated with improved local control in ES-SCLC patients with residual thoracic lesions following induction chemo-immunotherapy.

### 4.3 Safety of cTRT

The increased adverse events associated with cTRT following first-line immunotherapy are a significant concern. Currently, the safety data about the combination of immunotherapy with TRT in ES-SCLC patients predominantly originates from small samples or retrospective clinical studies. A phase I clinical study (65) conducted on ES-SCLC assessed the safety of pembrolizumab combined with TRT after induction chemotherapy. The findings indicated that patients treated with this combination had good tolerance, with only 6% experiencing grade 4 adverse events. A real-world study demonstrated that cTRT following first-line chemotherapy in conjunction with ICIs did not increase the incidence of adverse events (59). Another study (66) that combined results from three single-institution phase I/II trials demonstrated that cTRT combined with immunotherapy has acceptable safety and feasibility in the short term. Only three out of 53 patients who received cTRT (45Gy in 15 fractions) in combination with immunotherapy developed grade 3 or higher adverse events. In a retrospective study (57) of 36 patients with ES-SCLC, the majority underwent TRT (60Gy in 28 fractions) after first-line chemotherapy in conjunction with ICIs. Only 8% of these patients developed radiation-related pneumonitis, all of which were grade 1-2. Four patients discontinued immunotherapy due to immune-related pneumonitis but completed the planned cTRT treatment. However, in a real-world study (58) involving 211 ES-SCLC patients, 70 participants received TRT after first-line chemotherapy in conjunction with ICIs. The incidence of treatment-related pneumonitis in the TRT group significantly increased (P = 0.018), but most cases were grade 1 or 2. Overall, for ES-SCLC patients, cTRT would increase the incidence of treatment-related pneumonitis, but the grade is low and within a controllable stage. It is important to note that most current studies exploring the safety of cTRT combined with immunotherapy in ES-SCLC are single-center retrospective studies. The included patients have significant heterogeneity and selection bias, so the data should be interpreted with caution. Therefore, the reported acceptable toxicity profile may not be generalizable to all ES-SCLC patients. Larger, prospective studies are urgently needed to definitively establish the safety of this combination.

## 5 Conclusions

In the chemotherapy era, multiple retrospective and prospective studies have demonstrated that cTRT can improve survival in ES-SCLC patients who respond effectively to systemic treatment, particularly those with intrathoracic residuals and a lower extrathoracic tumor burden. However, as the first-line treatment for ES-SCLC transitions into the immunotherapy era, the value of cTRT has been widely debated. In the distinct immunosuppressive microenvironment of SCLC, the combination of radiotherapy and immunotherapy may enhance efficacy, but the potential for increased toxicity must be carefully considered. Multiple clinical studies have confirmed that cTRT is safe and feasible. The pattern of failure in the IMPOWER133 trial suggests that local treatment may offer potential benefits after first-line chemotherapy combined with immunotherapy for ES-SCLC. Multiple retrospective studies and several phase I and II prospective studies have preliminarily demonstrated the safety and effectiveness of cTRT for ES-SCLC. In the context of immunotherapy, future research should explore the selection of appropriate populations for thoracic radiotherapy, determine the optimal radiation dose and fractionation mode, and establish the timing for adding radiotherapy.
